# CXCL1 Promotes Fibrotic Remodeling in Atrial Fibrillation via Activation of TXNDC5 and Endoplasmic Reticulum Stress

**DOI:** 10.1155/cdr/7892499

**Published:** 2025-10-28

**Authors:** Ran Yin, Cheng-Long Wu, Si-Yang Yu, Kun Huang, Yuan Wen, Jun-Gang Nie, Ru Ying

**Affiliations:** Department of Cardiology, The First Affiliated Hospital of Nanchang University, Nanchang, China

**Keywords:** atrial fibrillation, atrial fibrosis, cardiac fibroblasts, CXCL1, endoplasmic reticulum stress, TXNDC5

## Abstract

**Background:**

Atrial fibrosis is a key structural substrate in atrial fibrillation (AF). This work was conducted to investigate the profibrotic effects of chemokine C-X-C motif Ligand 1 (CXCL1) and elucidate the actions of endoplasmic reticulum stress (ERS) and ER-resident protein thioredoxin domain-containing Protein 5 (TXNDC5) in this process.

**Methods:**

Serum CXCL1 concentrations were measured in patients with AF and healthy controls. The effects of CXCL1 on atrial fibrosis were evaluated using ex vivo rat atrial tissue culture. Additionally, the influence of CXCL1 on collagen synthesis, ERS activation, and TXNDC5 expression was assessed in primary rat cardiac fibroblasts. Pharmacological inhibition of ERS and gene silencing of TXNDC5 were employed to decipher underlying mechanisms.

**Results:**

CXCL1 levels were elevated in patients with AF compared to controls. In ex vivo rat atrial tissue, CXCL1 treatment induced marked fibrosis and upregulated the expression of ERS markers GRP78 and ATF6, as well as TXNDC5. In cardiac fibroblasts, CXCL1 promoted the secretion of Collagen I, Collagen III, and TGF-*β*1. Notably, both ERS inhibition and TXNDC5 knockdown effectively attenuated CXCL1-induced fibroblast activation and extracellular matrix protein expression.

**Conclusion:**

CXCL1 promotes atrial fibrosis through the activation of ERS and upregulation of TXNDC5, potentially contributing to atrial remodeling and the pathogenesis of AF. Targeting the CXCL1–ERS–TXNDC5 axis may offer a novel therapeutic approach for preventing and treating AF-related atrial fibrosis.

## 1. Introduction

Atrial fibrillation (AF) is a common arrhythmia among older individuals and a major contributor to adverse cardiovascular outcomes, including stroke, heart failure, and increased mortality risk [1]. The development of AF is closely associated with atrial electrical and structural remodeling, both of which play pivotal roles in establishing and maintaining the arrhythmogenic substrate [2–4]. Key pathological processes—such as oxidative stress, inflammation, and calcium overload—drive atrial myocardial fibrosis, facilitating the onset and persistence of AF [5, 6]. These processes increase the susceptibility of atrial tissue to fibrillation, while AF itself further exacerbates myocardial remodeling and fibrosis, forming a self-perpetuating vicious cycle that complicates treatment and heightens recurrence risk [7].

The chemokine C-X-C motif Ligand 1 (CXCL1) has been reported in fibrotic processes across multiple organ systems, including the lungs, liver, and kidneys [8–10]. Among various circulating biomarkers associated with AF, evidence points to a significant role for CXCL1's receptor—C-X-C motif chemokine Receptor 2 (CXCR2)—in promoting cardiac remodeling and fibrosis through the activation of the renin–angiotensin–aldosterone system (RAAS). CXCR2 has been shown to accelerate disease progression in cardiovascular disorders such as atherosclerosis and hypertension [11, 12]. In addition, CXCR2 facilitates monocyte infiltration into cardiac tissue, increasing susceptibility to RAAS-related AF in murine models [11]. By promoting the infiltration of neutrophils and monocytes, CXCR2 contributes to a heightened inflammatory response. However, whether CXCL1 itself directly contributes to atrial myocardial fibrosis and the mechanisms underlying this potential effect remain unclear.

Endoplasmic reticulum stress (ERS) is increasingly recognized as a critical contributor to cardiac fibrosis. The thioredoxin domain-containing Protein 5 (TXNDC5), regulated by the ERS-related activating transcription Factor 6 (ATF6) pathway, exerts essential functions in fibrotic remodeling by promoting proper folding and secretion of extracellular matrix (ECM) proteins within the endoplasmic reticulum [13]. Under hypoxic and inflammatory conditions, ERS leads to the activation and nuclear translocation of ATF6, which in turn upregulates TXNDC5 transcription. The resulting increase in TXNDC5 expression enhances ECM protein synthesis and accelerates fibrotic progression [13–15].

Given the potential involvement of CXCL1 and the ERS-associated protein TXNDC5 in atrial fibrosis, we aimed to investigate their interrelationship and underlying mechanisms. This study sought to elucidate the actions of CXCL1 and TXNDC5 in AF pathogenesis, with the goal of identifying novel targets for predicting and managing AF.

## 2. Materials and Methods

### 2.1. Patient Recruitment and Sample Collection

This single-center study was conducted at the First Affiliated Hospital of Nanchang University and approved by the institutional ethics committee of the First Affiliated Hospital of Nanchang University (Ethics Code: [2021] Medical Research Ethics Review No. 12-012). Between September and December 2020, patients with AF and patients with paroxysmal supraventricular tachycardia (PSVT), all without comorbid cardiovascular diseases, were recruited. All participants underwent radiofrequency ablation and atrial septal puncture. Blood samples were collected from the peripheral circulation, left atrium, and right atrium during the procedure. The cohort included 29 patients with AF and 16 with PSVT.

To validate these findings, an independent cohort comprising 65 patients with AF and 150 healthy controls was also enrolled. Only peripheral blood samples were obtained from this validation group. Diagnoses of AF and PSVT were based on standard 12-lead electrocardiography (ECG) or Holter ECG monitoring.

### 2.2. Blood Sample Processing

During ablation, 2 mL of blood was collected from each of the three anatomical sites using vacuum tubes containing anticoagulant and separator gel. Samples were temporarily stored at 4°C, then centrifuged at 4000 rpm (relative centrifugal force: 2680 × g) for 10 min using a TG16W centrifuge (Changsha Xiangzhi Centrifuge Instrument Co. Ltd., Changsha, China). The resulting serum was aliquoted and stored at −80°C until further analysis. Peripheral blood samples from the validation cohort were processed identically.

### 2.3. Enzyme-Linked Immunosorbent Assay (ELISA)

Serum CXCL1 concentrations were quantified using a human CXCL1 ELISA kit (MM-1850H2, Jiangsu Meimian Industrial Co. Ltd., Jiangsu, China), according to the manufacturer's protocol.

### 2.4. Ex Vivo Atrial Tissue Culture

Left atrial tissues were harvested from 8-week-old Sprague–Dawley rats (GemPharmatech LLC, China) under anesthesia with intraperitoneal pentobarbital (80 mg/kg). The tissues were immediately transferred to CO_2_-independent cryogenic culture medium. Tissue explants were placed endocardial side down on 0.4 *μ*m porous polyester membrane inserts (Millicell, Merck Millipore, Darmstadt, Germany) in culture medium supplemented with insulin, transferrin, sodium selenite, 5% fetal bovine serum (FBS), glucose, isoproterenol (1 nmol/L), and penicillin/streptomycin (100 U/mL) (Gibco, Invitrogen, Grand Island, New York, United States) [16]. After preincubation at 37°C in 5% CO_2_ for 15 min, 20 *μ*L of either CXCL1-containing medium (5 *μ*g/L) or control medium was applied daily to the epicardial surface for 7 consecutive days.

### 2.5. Histological Analysis

After treatment, atrial tissues were fixed in formalin, paraffin-embedded, and sectioned at 5 *μ*m. Sections were stained with hematoxylin and eosin (H&E) and Masson's trichrome (JL18211, Laibio, Shanghai, China) to assess fibrosis. Images were acquired using a BZ-9000 BioRevo fluorescence microscope (Keyence, Osaka, Japan) at 40× and 200× magnifications. Fibrosis was quantified by calculating the ratio of fibrotic to nonfibrotic myocardial area, and the mean percentage of fibrosis was reported.

### 2.6. Immunohistochemistry for *α*-SMA

Immunohistochemical staining for *α*-SMA was performed on deparaffinized atrial sections. After blocking with phosphate-buffered saline (PBS) containing 1% bovine serum albumin for 30 min, sections were incubated overnight at 4°C with rabbit anti-*α*-SMA antibody (ab5694, Abcam, 1:3200 dilution). After rinsing with PBS, sections were incubated with a secondary antibody for 2 h at room temperature and visualized with 3,3⁣′-diaminobenzidine for 7 min [13]. The quantification of the *α*-SMA was performed by using ImageJ software, and the vascular staining was excluded from the analysis.

### 2.7. Primary Cardiac Fibroblast Culture, Short Hairpin RNA (shRNA) Transfection, and Drug Treatment

Cardiac fibroblasts were isolated from 1–3-day-old Sprague–Dawley neonatal rats. Hearts were excised, minced in PBS, and digested with 2 mg/mL trypsin. After centrifugation at 240 × g for 3 min, the cell pellet was resuspended in DMEM containing 5% FBS, penicillin (200 U/mL), streptomycin (200 *μ*g/mL), and vitamin B12 (2 *μ*g/mL) and cultured at 37°C in 5% CO_2_. Upon confluence, the cells were passaged using trypsin, and second passage fibroblasts were used for experiments.

shRNAs targeting TXNDC5 genes were constructed using the pLKO.1 vector. Lentivirus production involved transfecting shRNA into 293T cells using PEI transfection reagent, collecting lentivirus at 72 h posttransfection. Cardiac fibroblasts were transfected with synthetic lentivirus. The stably transfected cardiac fibroblasts were subsequently screened with 3 *μ*g/mL puromycin (Sigma-Aldrich), and related experiments were subsequently performed.

For the CXCL1 treatment, cells were treated with 5 *μ*g/L CXCL1 for 72 h. For the 4-phenylbutyrate (4-PBA) treatment, cells were pretreated with 5 mM 4-PBA for 0.5 h, followed by incubating with 5 *μ*g/L CXCL1 for another 72 h. Cells were processed for subsequent assays.

### 2.8. Western Blot

Protein expression of fibrosis-related markers (Collagen I, Collagen III, and TGF-*β*1) and ER stress proteins (GRP78, ATF6, and TXNDC5) was evaluated by western blot. Equal amounts of protein were separated by SDS-PAGE and transferred onto polyvinylidene fluoride (PVDF) membranes. Membranes were blocked with 2% BSA and incubated overnight at 4°C with primary antibodies against Collagen I (ab260043, 1:1000), Collagen III (ab7778, 1:5000), TGF-*β*1 (ab215715, 1:1000), GRP78 (ab108615, 1:2000), ATF6 (ab203119, 1:1000), and TXNDC5 (bs-7579R, Bioss, 1:1000). Following PBS washes, membranes were incubated with HRP-conjugated secondary antibodies at 37°C, and signals were visualized using enhanced chemiluminescence. GAPDH was used as the loading control. The uncropped images of the western blot results were provided as Figures S1, S2, and S3.

### 2.9. Quantitative Real-Time PCR (qRT-PCR)

Total RNA was extracted from rat atrial tissue using the RNeasy Kit (QIAGEN, Hilden, Germany), and cDNA was synthesized using the Transcript First Strand cDNA Synthesis Kit (Roche Diagnostics, Mannheim, Germany). PCR amplification was performed using the following thermal profile: 95°C for 3 min, followed by 40 cycles of 95°C for 30 s, 58°C for 30 s, and 72°C for 20 s. Relative gene expression was determined using the 2^−*ΔΔ*Ct^ method with GAPDH as the internal reference. Primer sequences used are listed in Table 1. Expression levels in the CXCL1 group were normalized to the control group.

### 2.10. Statistical Analyses

Continuous variables are presented as mean ± standard deviation (SD), while categorical variables are reported as counts and percentages. Comparisons of CXCL1 levels between patient groups at the same anatomical site were conducted using unpaired *t*-tests. Differences in CXCL1 concentrations across the three anatomical sites within the same patient group were analyzed by one-way ANOVA. Categorical variables were compared using the *χ*^2^ test. A two-tailed *p* value of < 0.05 was considered statistically significant.

## 3. Results

### 3.1. Baseline Characteristics of Study Participants

Baseline demographic and clinical characteristics of the AF and control groups are summarized in Table 2, with additional data from the supplemental cohort presented in Table 3. No differences in most baseline parameters were observed between the two groups. However, as expected, the anteroposterior diameter of the left atrium was significantly larger in patients with AF compared to controls, consistent with the known pathophysiological remodeling associated with AF. Although the control group was on average younger than the AF group, there is currently no evidence indicating that CXCL1 expression is age-dependent.

### 3.2. CXCL1 Expression Is Elevated in AF Patients

**Figure 1:**
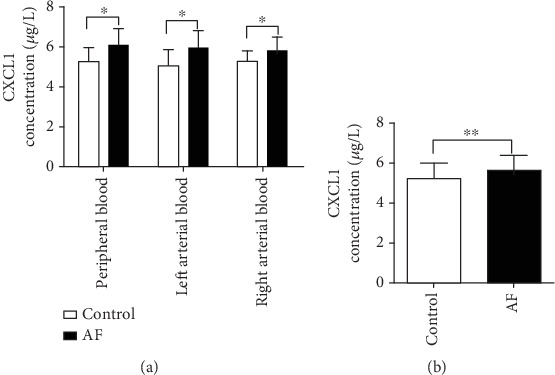
Serum CXCL1 levels in patients with AF and healthy controls. (a) Comparison of CXCL1 concentrations measured from three anatomical sites—peripheral blood, left atrial blood, and right atrial blood—in patients with AF and control subjects. (b) Validation of CXCL1 levels in peripheral blood from an independent supplemental cohort of AF patients and controls. Data are presented as mean ± SD. ⁣^∗^*p* < 0.05 and ⁣^∗∗^*p* < 0.01 versus control group (unpaired test).

CXCL1 concentrations were significantly higher in patients with AF across all three sampling sites—peripheral blood, left atrium, and right atrium—compared to controls (Figure 1a). Specifically, peripheral blood CXCL1 levels in the AF group were 6.062 ± 0.862 versus 5.267 ± 0.717 *μ*g/L in controls (*p* = 0.003). In the left atrial blood, concentrations were 5.952 ± 0.849 *μ*g/L in the AF group versus 5.070 ± 0.802 *μ*g/L in controls (*p* = 0.001), and in the right atrial blood, levels were 5.773 ± 0.749 versus 5.248 ± 0.554 *μ*g/L, respectively (*p* = 0.018).

Within-group comparisons revealed no differences in CXCL1 levels among three anatomical sites in either AF (*p* = 0.405) or control groups (*p* = 0.680), indicating a systemic elevation rather than site-specific expression. These findings were corroborated in the validation cohort, where CXCL1 levels in peripheral blood were higher in patients with AF than in healthy controls (Figure 1b).

### 3.3. CXCL1 Promotes Atrial Fibrosis and Upregulation of *α*-SMA in Ex Vivo Tissue

**Figure 2:**
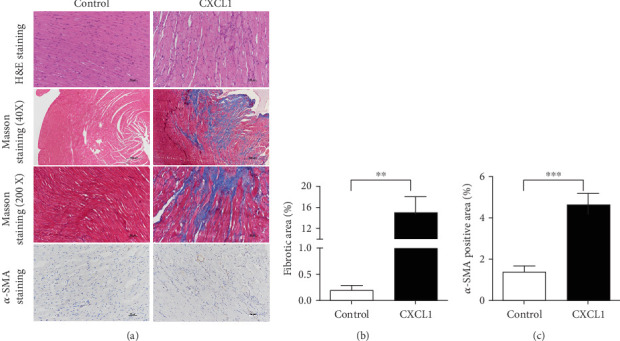
CXCL1 promotes atrial fibrosis and *α*-SMA expression in ex vivo rat atrial tissue. (a) Representative histological images showing hematoxylin and eosin (HE) staining, Masson's trichrome staining (40× and 200× magnification), and immunohistochemistry for *α*-SMA following CXCL1 treatment. (b) Quantification of fibrotic area (%) based on Masson's staining, analyzed using ImageJ software. (c) Quantification of *α*-SMA-positive area (%) using Image-Pro Plus software. Data are expressed as mean ± SD. ⁣^∗∗^*p* < 0.01 and ⁣^∗∗∗^*p* < 0.001 versus control group.

Ex vivo exposure of isolated rat atrial tissue to CXCL1 (5 *μ*g/L) for 7 days resulted in a marked increase in fibrotic area, as determined by Masson's trichrome staining. Additionally, immunohistochemistry revealed an increase in *α*-SMA expression, a marker of myofibroblast activation, in the CXCL1-treated group compared to controls (Figures 2a, 2b, and 2c). Quantitative analysis confirmed that both fibrotic area and *α*-SMA-positive staining were significantly elevated in the CXCL1 group (⁣^∗^*p* < 0.05).

### 3.4. CXCL1 Stimulates ECM Protein Secretion in Cardiac Fibroblasts

**Figure 3:**
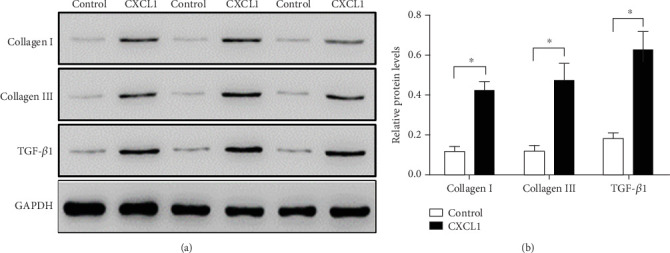
CXCL1 promotes extracellular matrix protein expression in rat cardiac fibroblasts. (a) Representative western blot images of Collagen I (139 kDa), Collagen III (138 kDa), and TGF-*β*1 (44 kDa) following 72-h stimulation with 5 *μ*g/L CXCL1. (b) Densitometric analysis of western blot bands quantified using ImageJ software. Data are shown as mean ± SD. ⁣^∗^*p* < 0.05 versus control group. All experiments were performed in triplicate.

To assess the profibrotic effects of CXCL1 at the cellular level, primary rat cardiac fibroblasts were stimulated with 5 *μ*g/L CXCL1 for 72 h. Western blot analysis showed significantly increased expression of ECM proteins, including Collagen I, Collagen III, and TGF-*β*1, in CXCL1-treated cells compared with controls (Figure 3a,b, ⁣^∗^*p* < 0.05). These results confirm that CXCL1 promotes fibrogenic activation in cardiac fibroblasts.

### 3.5. CXCL1 Induces ERS in Fibroblasts

**Figure 4:**
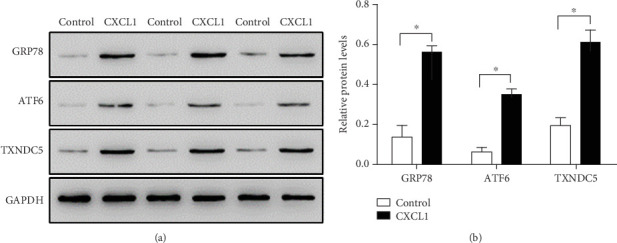
CXCL1 induces ERS markers in rat cardiac fibroblasts. (a) Representative western blot showing increased expression of GRP78 (72 kDa), ATF6 (75 kDa), and TXNDC5 (44 kDa) following stimulation with 5 *μ*g/L CXCL1. (b) Quantitative analysis of protein expression using ImageJ software. Data represent mean ± SD. ⁣^∗^*p* < 0.05 versus control group. All experiments were repeated three times.

Western blot analysis further demonstrated that CXCL1 significantly increased the expression of key ERS markers, including GRP78, ATF6, and TXNDC5, in rat cardiac fibroblasts (Figure 4a,b, ⁣^∗^*p* < 0.05). These findings suggest that CXCL1-induced ER stress may contribute to its profibrotic effects.

### 3.6. ERS Inhibition and TXNDC5 Silencing Reversed CXCL1-Induced Fibrogenic Signaling

**Figure 5:**
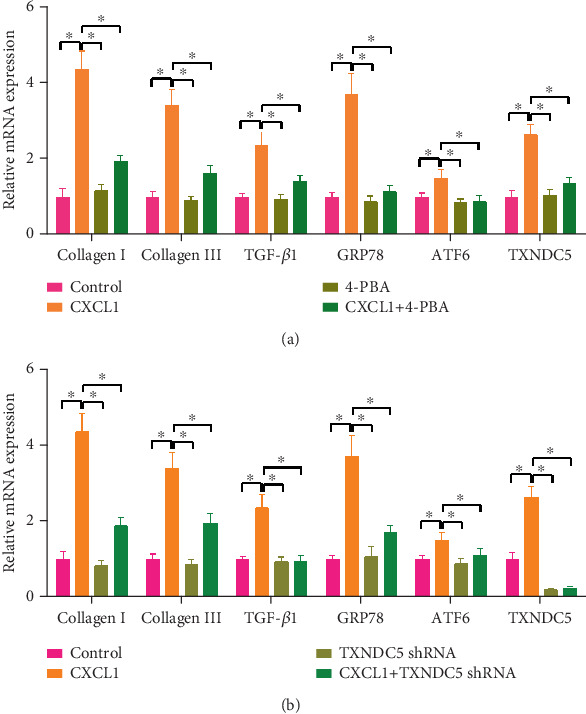
Reversal of CXCL1-induced mRNA expression by ERS inhibition and TXNDC5 knockdown. (a) Relative mRNA expression levels of Collagen I (Col I), Collagen III (Col III), TGF-*β*1, GRP78, ATF6, and TXNDC5 in control, CXCL1-treated, 4-PBA-treated, and CXCL1 + 4-PBA cotreated groups. (b) mRNA expression levels of the same genes in control, CXCL1-treated, TXNDC5 shRNA-treated, and CXCL1 + TXNDC5 shRNA cotreated groups. Data are shown as mean ± SD. Significant differences between groups were indicated as ⁣^∗^*p* < 0.05. All experiments were conducted in triplicate.

**Figure 6:**
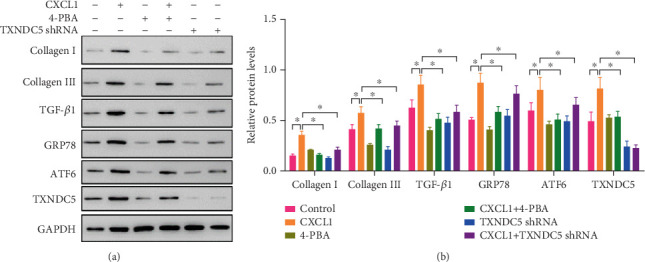
Protein-level reversal of CXCL1-induced fibrosis markers by 4-PBA and TXNDC5 shRNA. (a) Representative western blot showing expression of Collagen I (139 kDa), Collagen III (138 kDa), TGF-*β*1 (44 kDa), GRP78 (72 kDa), ATF6 (75 kDa), and TXNDC5 (44 kDa) across treatment groups: control, CXCL1, 4-PBA, CXCL1 + 4-PBA, TXNDC5 shRNA, and CXCL1 + TXNDC5 shRNA. (b) Densitometric quantification of protein expression levels. Values are presented as mean ± SD. Significant differences between groups were indicated as ⁣^∗^*p* < 0.05. All experiments were repeated three times.

To confirm ERS's involvement in CXCL1-induced fibrosis, we evaluated the actions of 4-PBA, a chemical chaperone, and ERS inhibitor. As shown in Figure 5a, 4-PBA significantly attenuated CXCL1-induced mRNA expression of Collagen I, Collagen III, TGF-*β*1, GRP78, ATF6, and TXNDC5 (⁣^∗^*p* < 0.05). Similarly, protein levels of these markers were also suppressed by 4-PBA, as demonstrated in Figure 6a,b.

Furthermore, targeted knockdown of TXNDC5 using shRNA effectively reduced both mRNA and protein levels of fibrogenic and ERS-related markers that were upregulated by CXCL1 (Figures 5b and 6a,b, ⁣^∗^*p* < 0.05). These data strongly indicate that TXNDC5 is a critical effector of the ERS pathway involved in CXCL1-mediated fibrosis.

These findings support a mechanistic link between CXCL1 signaling, ERS activation, and fibrotic remodeling in atrial tissue. Inhibition of ER stress or silencing of TXNDC5 significantly mitigates CXCL1-induced profibrotic responses, highlighting potential therapeutic targets in the prevention and treatment of atrial fibrosis.

## 4. Discussion

Atrial remodeling, particularly structural remodeling characterized by fibrosis, is a key contributor to the onset and progression of AF and is strongly influenced by proinflammatory cytokines and chemokines [17–19]. Our work identified a novel mechanistic link between the chemokine CXCL1 and atrial fibrosis, implicating ERS and the ER-resident protein TXNDC5 as downstream effectors of CXCL1-mediated fibrogenesis. Collectively, our findings offer new insights into the molecular pathways driving atrial fibrosis in AF, independent of other cardiovascular comorbidities. Our clinical data demonstrated significantly elevated levels of CXCL1 in patients with AF compared to controls, across all three sampled compartments. However, there were no significant differences in CXCL1 concentrations among the three sites within either group (*p* > 0.05), suggesting a systemic rather than localized elevation of this chemokine in AF. These observations were further validated by data from a supplemental cohort, confirming the robustness of our results. Using an ex vivo model, we further demonstrated that CXCL1 exposure directly promoted atrial fibrosis, as evidenced by increased collagen deposition and upregulation of *α*-SMA in rat atrial tissue. At the cellular level, CXCL1 stimulated cardiac fibroblasts to express higher levels of Collagen I, Collagen III, and TGF-*β*1—hallmark proteins of ECM remodeling. This fibrogenic response was accompanied by marked upregulation of ERS markers, including GRP78, ATF6, and TXNDC5, supporting a mechanistic role for ER stress in CXCL1-induced fibrosis. Importantly, pharmacological inhibition of ERS using 4-PBA reversed the upregulation of ECM-related and ERS-related genes and proteins, indicating that the profibrotic effect of CXCL1 is mediated, at least in part, via ER stress signaling. Furthermore, silencing TXNDC5 using shRNA also attenuated CXCL1-induced increases in collagen and TGF-*β*1 expression, reinforcing the pivotal role of the ATF6-TXNDC5 axis in this process. Notably, TXNDC5 knockdown alone was sufficient to suppress fibroblast activation, highlighting its function as a critical downstream mediator of ERS-driven fibrosis and as a potential therapeutic target.

CXCL1 is well-established as a potent chemoattractant that facilitates neutrophil and monocyte infiltration, amplifying the local inflammatory response [20–22]. Prior studies have linked CXCL1 to atrial fibrosis in hypertensive models, largely through its role in enhancing inflammation and oxidative stress, thereby promoting RAAS-related structural remodeling [23–25]. However, these studies did not address the impact of CXCL1 in patients with isolated AF without underlying cardiovascular disease. Our findings extend this understanding by showing that CXCL1 itself is sufficient to induce atrial fibrosis via ERS and TXNDC5 activation, independent of other comorbidities.

**Figure 7:**
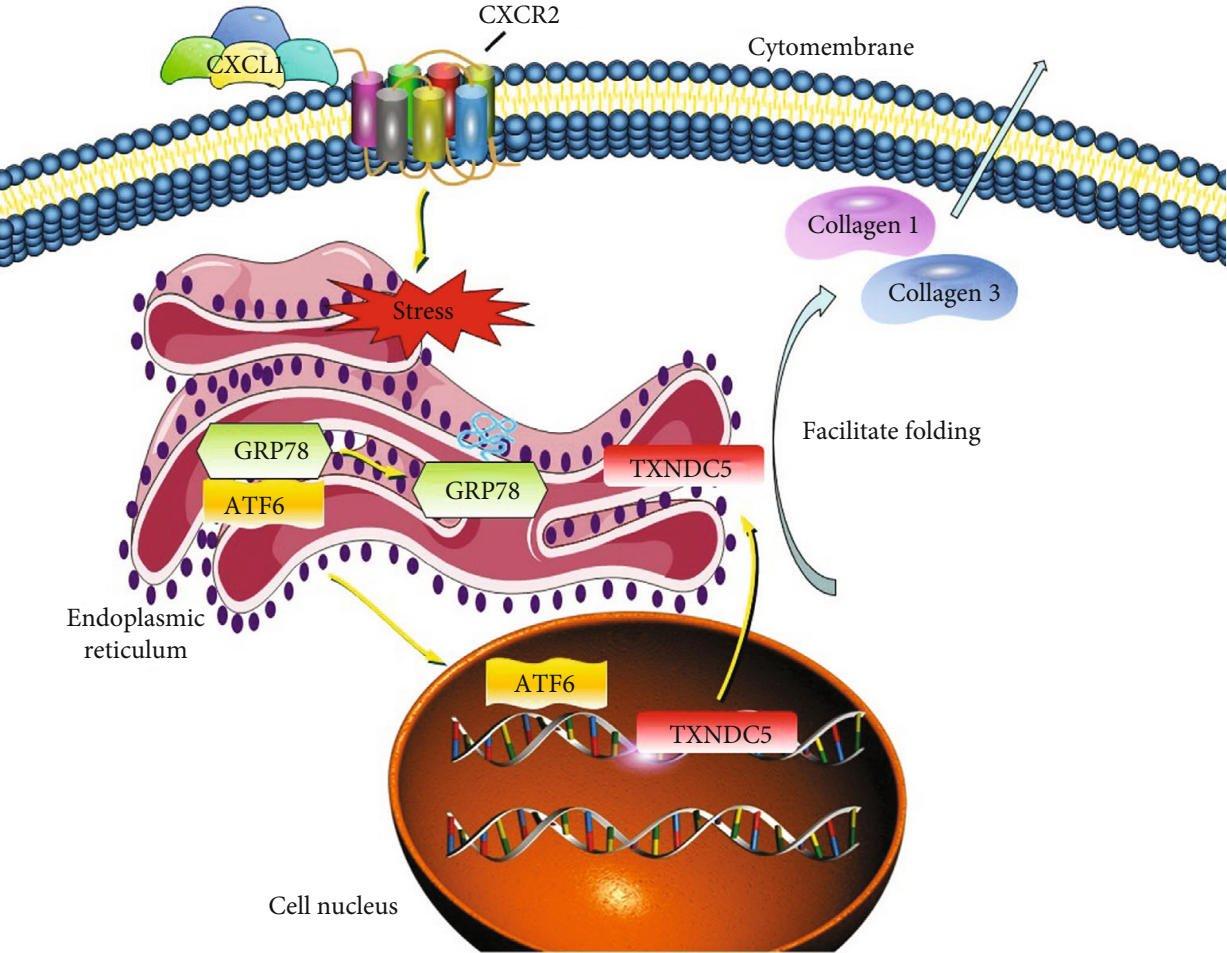
Proposed mechanistic model of CXCL1-induced atrial fibrosis. Schematic illustration of the pathway through which CXCL1 promotes atrial fibrosis via ERS activation and upregulation of TXNDC5. Activation of ATF6 leads to nuclear transcription of TXNDC5, enhancing collagen folding and secretion, thereby contributing to atrial remodeling and increased susceptibility to atrial fibrillation.

The ER stress response in fibroblasts involves the dissociation of GRP78 from ATF6, enabling ATF6 to translocate into the nucleus and upregulate TXNDC5. TXNDC5, in turn, facilitates the proper folding and secretion of ECM proteins such as Collagens I and III [13, 26–28]. This pathway provides a plausible mechanism through which CXCL1 promotes atrial fibrosis at the molecular level. We propose that CXCL1 may act in an endocrine or paracrine manner to initiate this fibrotic cascade, leading to irreversible atrial remodeling and increased arrhythmogenic susceptibility. A proposed schematic of this mechanism is illustrated in Figure 7.

Our study has some limitations, particularly a small sample size in the early stage and the absence of further subdivision of AF types. Besides, 4-PBA has been widely recognized as a modulator of ER stress; its influence on downstream gene expression and the production of secretory proteins remains controversial [29, 30]. Additionally, limited membrane penetration and inefficient intracellular delivery to the intended subcellular compartment represent significant limitations of 4-PBA [31]. While we validated the CXCL1–ERS–TXNDC5 axis in cellular models, direct functional validation in atrial tissues, such as applying 4-PBA treatment and measuring pathway-specific RNA/protein expression, was not performed due to funding and time constraints, limiting the translation of in vitro mechanisms to in vivo physiological contexts. The clinical cohort of the present study was relatively small [29] AF patients and 16 controls), with initially limited baseline characteristics in the validation cohort; this small sample size precluded robust statistical analyses to explore correlations between CXCL1 levels and AF severity, progression, or phenotypic subtypes, potentially obscuring the biomarker potential of CXCL1. The mechanistic investigation remains incomplete, as we did not fully characterize upstream receptors (e.g., CXCR1/CXCR2) mediating CXCL1-induced ERS or downstream effects of TXNDC5 on cardiomyocyte apoptosis/fibrosis, nor did we explore interactions with other AF-related pathways or evaluate in vivo drug interventions targeting this axis. These limitations highlight the need for future studies with larger clinical cohorts, tissue-specific functional experiments, and animal models to fully validate and expand the role of the CXCL1–ERS–TXNDC5 axis in atrial remodeling and AF pathogenesis.

## 5. Conclusion

This study demonstrates that CXCL1 promotes atrial ECM deposition through the activation of the ER stress protein TXNDC5, thereby facilitating atrial fibrosis and enhancing susceptibility to AF. These findings identify CXCL1 and TXNDC5 as potential biomarkers and therapeutic targets in AF-associated atrial remodeling.

## Figures and Tables

**Table 1:** qRT-PCR primers for the quantification of *Rattus norvegicus* gene expression.

**Gene**	**Forward primer**	**Reverse primer**
Collagen 1	5⁣′-TCCTGGCAAGAACGGAGAT-3⁣′	5⁣′-CAGGAGGTCCACGCTCAC-3⁣′
Collagen 3	5⁣′-GAGGAATGGGTGGCTATCCT-3⁣′	5⁣′-GGTATCCAGGAGAACCAGGAG-3⁣′
TGF-ꞵ1	5⁣′-CCTGGAAAGGGCTCAACAC-3⁣′	5⁣′-TGCCGTACACAGCAGTTCTT-3⁣′
GRP78	5⁣′-ACATGGACCTGTTCCGCTCTA-3⁣′	5⁣′-TGGCTCCTTGCCATTGAAGA-3⁣′
ATF6	5⁣′-GATGCAGCACATGAGGCTTA-3⁣′	5⁣′-CAGGAACGTGCTGAGTTGAA-3⁣′
TXNDC5	5⁣′-CTCTGGGCCTTGAACATT-3⁣′	5⁣′-CCCTCAGTGACTCCAAA-3⁣′

**Table 2:** Comparison of baseline data between the control group and AF group.

	**Control group (** **n** = 16**)**	**AF group (** **n** = 29**)**	**p** ** value**
Age (years)	53.0 ± 14.9	62.4 ± 12.1	0.027^∗^
Male (*n* [%])	12 (75.0)	17 (58.6)	0.272
EF (%)	63.3 ± 3.7	60.8 ± 6.6	0.176
Anteroposterior diameter of the left atrium (mm)	30.3 ± 3.2	37.5 ± 6.5	<0.001^∗∗^
ALT (U/L)	28.2 ± 19.6	24.5 ± 12.8	0.449
AST (U/L)	28.0 ± 11.9	25.8 ± 10.1	0.526
Cr (*μ*mol/L)	105.0 (51, 78.1)	71.9 (63.1, 82.5)	0.297
Ura (*μ*mol/L)	351.4 ± 89.4	403.7 ± 86.8	0.062
TG (mmol/L)	1.7 ± 1.1	1.5 ± 0.9	0.69
TC (mmol/L)	3.9 ± 1.6	4.3 ± 1.2	0.423
HDL (mmol/L)	1.1 ± 0.4	1.2 ± 0.3	0.293
LDL (mmol/L)	2.6 ± 1.0	2.6 ± 0.9	0.996
K (mmol/L)	4.1 ± 0.4	4.2 ± 0.4	0.667
Na (mmol/L)	142.0 ± 2.1	141.7 ± 2.5	0.68
Lymphocyte (⁣^∗^10^9^/L)	1.6 ± 0.7	1.6 ± 0.6	0.847
Neutrophil (⁣^∗^10^9^/L)	3.3 ± 1.1	2.9 ± 1.3	0.153

⁣^∗^*p* < 0.05; ⁣^∗∗^*p* < 0.01

**Table 3:** Comparison of baseline data between the control group and the AF group (supplemental study).

	**Control group (** **n** = 150**)**	**AF group (** **n** = 65**)**	**p** ** value**
Age (years)	53.0 ± 14.9	62.4 ± 12.1	<0.001^∗∗^
Male (*n* [%])	12 (75.0)	17 (58.6)	0.235
EF (%)	63.3 ± 3.7	60.8 ± 6.6	0.012^∗^
Anteroposterior diameter of the left atrium (mm)	30.3 ± 3.2	37.5 ± 6.5	<0.001^∗∗^
ALT (U/L)	28.2 ± 19.6	24.5 ± 12.8	0.530
AST (U/L)	28.0 ± 11.9	25.8 ± 10.1	0.016^∗^
Cr (*μ*mol/L)	105.0 (51, 78.1)	71.9 (63.1, 82.5)	0.166
Ura (*μ*mol/L)	351.4 ± 89.4	403.7 ± 86.8	0.796
TG (mmol/L)	1.7 ± 1.1	1.5 ± 0.9	0.018^∗^
TC (mmol/L)	3.9 ± 1.6	4.3 ± 1.2	<0.001^∗∗^
HDL (mmol/L)	1.1 ± 0.4	1.2 ± 0.3	0.011^∗^
LDL (mmol/L)	2.6 ± 1.0	2.6 ± 0.9	<0.001^∗∗^
K (mmol/L)	4.1 ± 0.4	4.2 ± 0.4	0.242
Na (mmol/L)	142.0 ± 2.1	141.7 ± 2.5	0.312
Lymphocyte (⁣^∗^10^9^/L)	1.6 ± 0.7	1.6 ± 0.6	0.034^∗^
Neutrophil (⁣^∗^10^9^/L)	3.3 ± 1.1	2.9 ± 1.3	0.937

⁣^∗^*p* < 0.05; ⁣^∗∗^*p* < 0.01

## Data Availability

Data are available on reasonable request.

## Supporting Information

Supporting Information Additional supporting information can be found online in the Supporting Information section. The uncropped images of the western blot results are provided as Figures S1, S2, and S3.
