# Supplementary material for: CXCL1 Promotes Fibrotic Remodeling in Atrial Fibrillation via Activation of TXNDC5 and Endoplasmic Reticulum Stress
Source: Cardiovasc Ther. 2025 Oct 28;2025:7892499. doi: 10.1155/cdr/7892499 (PMC12585787; doi:10.1155/cdr/7892499)
Supplement: Supporting Information — Additional supporting information can be found online in the Supporting Information section. The uncropped images of the western blot results are provided as Figures S1, S2, and S3. [file 7892499.f1.pdf]

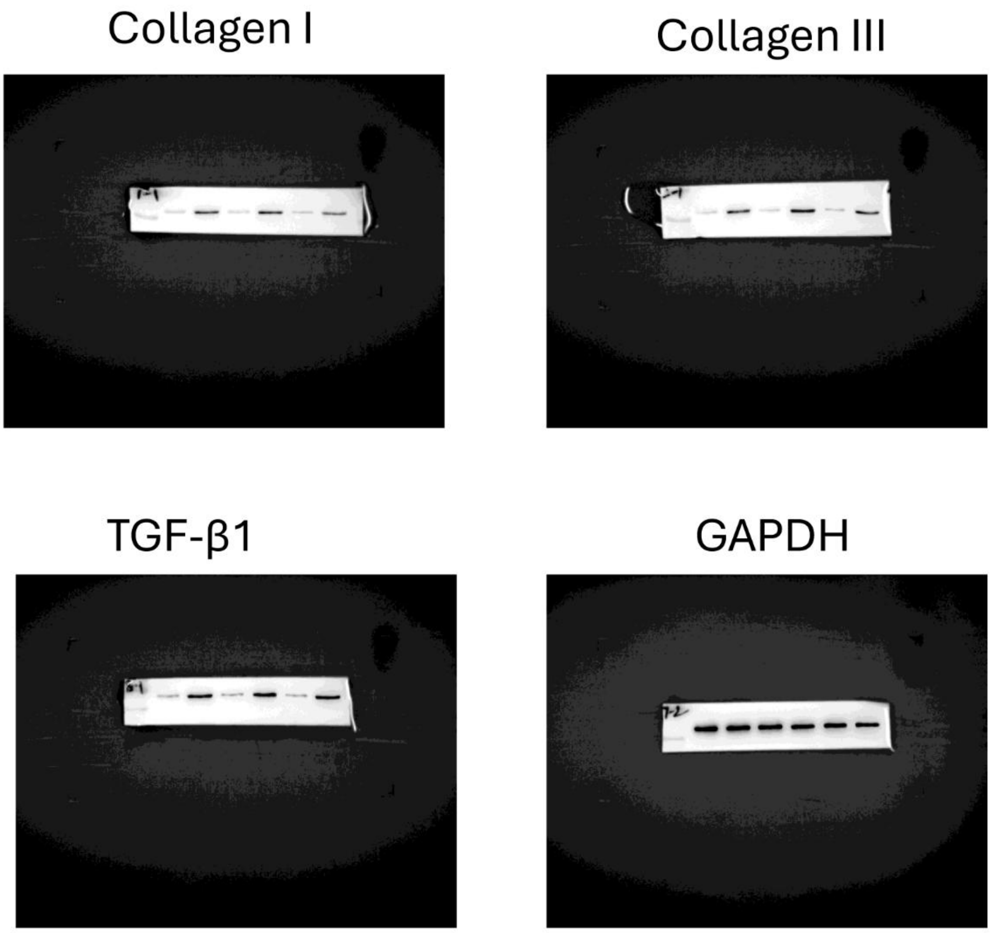

Supplemental Figure S1. Uncropped western blot images for Figure 3A.

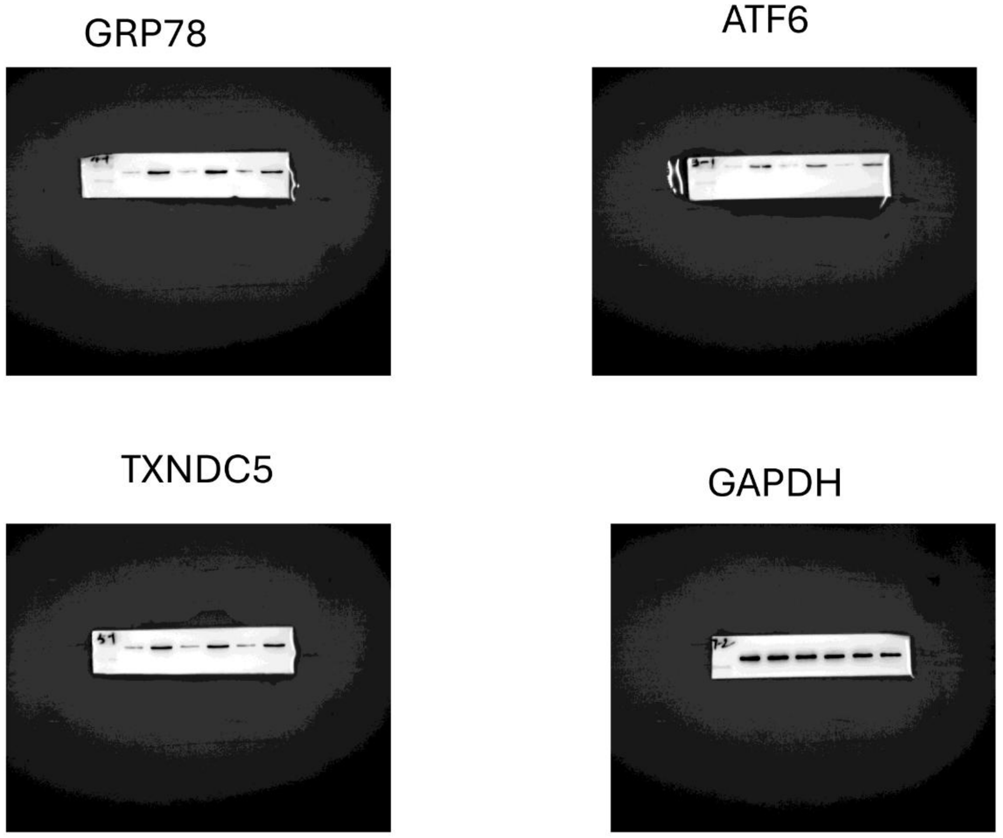

Supplemental Figure S2. Uncropped western blot images for Figure 4A.

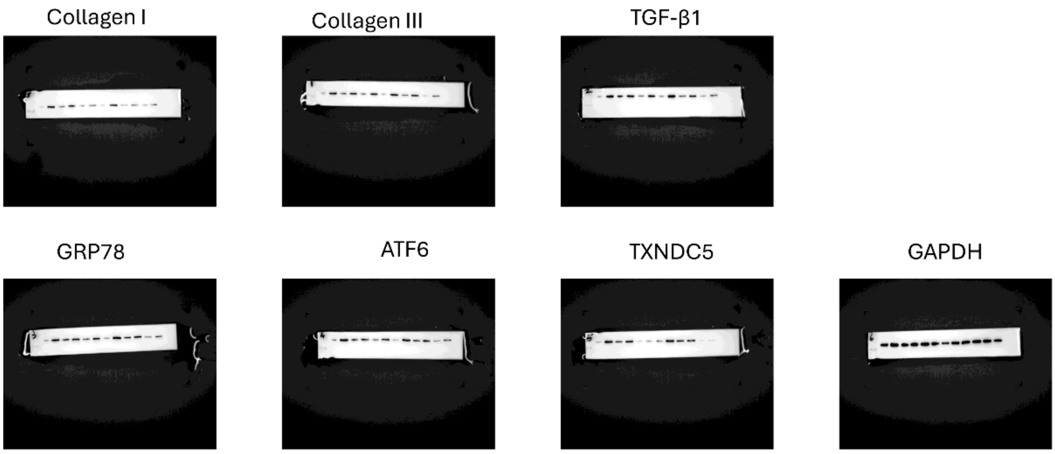

Supplemental Figure S3. Uncropped western blot images for Figure 6A.
